# Prevalence of chronic diseases among older patients in German general practices

**DOI:** 10.3205/000230

**Published:** 2016-03-03

**Authors:** Louis Jacob, Jessica Breuer, Karel Kostev

**Affiliations:** 1 Department of Biology, École Normale Supérieure de Lyon, Lyon, France; 2 Fresenius University of Applied Sciences, Frankfurt, Germany; 3 IMS Health, Epidemiology, Frankfurt, Germany

**Keywords:** prevalence of chronic diseases, older patients, Germany

## Abstract

**Aims:** To evaluate the prevalence of chronic diseases (CDs) among older patients in German general practices (GPs).

**Methods:** A total of 840,319 patients older than 65 years (359,289 male and 481,030 female) who consulted a GP between January and December 2014 were selected. Ten different CDs were considered: hypertension, lipid metabolism, diabetes, coronary heart disease, cancer, chronic obstructive pulmonary disease, heart failure, stroke, chronic kidney disease and osteoporosis. The prevalence, defined as the proportion of patients diagnosed with these disorders, was estimated.

**Results:** All CDs were very common in older subjects. Hypertension was the most common CD, affecting 65.7% of men and 66.1% of women. Stroke was the least frequent CD, with 6.6% of men and 5.1% of women displaying this condition. More than one out of two subjects had between one and three CDs (men: 57.7% and women: 59.3%). Approximately 25% of subjects had four or more CDs (men: 26.6% and women: 23.6%).

**Conclusions:** Our study showed that the prevalence of CDs is high in the German elderly population. Hypertension was the most frequent chronic condition and around 25% of patients displayed at least four CDs.

## Zusammenfassung

**Hintergrund:** Das Ziel dieser Studie ist es, die Häufigkeit von chronischen Erkrankungen bei älteren Patienten in deutschen hausärztlichen Praxen zu ermitteln.

**Methoden:** Es wurden insgesamt 840.319 Patienten im Alter über 65 Jahre (359.289 Männer und 481.030 Frauen) mit mindestens einer Visite beim Hausarzt zwischen Januar und Dezember 2014 identifiziert. Zehn unterschiedliche chronische Erkrankungen wurden untersucht: Hypertonie, Hyperlipidämie, Diabetes, koronare Herzkrankheit (KHK), Krebs, chronisch obstruktive Lungenerkrankung (COPD), Herzinsuffizienz, Schlaganfall, Niereninsuffizienz und Osteoporose. Prävalenz wurde definiert als der Anteil der Patienten mit der jeweiligen Diagnose.

**Ergebnisse:** Alle untersuchten Diagnosen waren häufig bei älteren Patienten. Am häufigsten wurde die Hypertonie diagnostiziert (65,7% der Männer und 66,1% der Frauen). Am seltensten war die Schlaganfall-Diagnose (6,6% der Männer und 5,1% der Frauen). 57,7% der Männer und 59,3% der Frauen hatten zwischen zwei und drei unterschiedliche chronische Erkrankungen; ca. 25% hatten vier oder mehr der untersuchten Diagnosen.

**Schlussfolgerungen:** In dieser Studie konnte eine hohe Prävalenz der chronischen Erkrankungen bei älteren Patienten gezeigt werden. Dabei wurde Hypertonie am häufigsten diagnostiziert und ca. 25% hatten mindestens vier der untersuchten Erkrankungen.

## Introduction

The proportion of people aged over 65 in Europe is increasing rapidly and scientists estimate that this age group will represent 29.5% of the total population by 2060, increasing from 17.4% in 2010 [1]. Low birth rates, steady increases in life expectancy, and the aging of Europeans born during the post-World War II baby boom are the main causes of this demographic change being experienced in this area of the world [1], [2]. The highest proportions of people over 65 in Europe are found in Germany and Italy, where numbers have doubled in less than sixty years to exceed 21% in 2014 [3]. As a result, these two countries are facing important challenges, particularly in the fields of medicine and health. 

One major consequence of population aging is an increase in the prevalence of chronic diseases (CDs), as age is known to be a non-modifiable risk factor for several of these disorders [4], [5], [6]. This increase in the number of patients with CDs is problematic for Europe, and particularly for Germany, since these noncommunicable diseases are already the leading cause of mortality. Indeed, cardiovascular diseases and cancers are responsible for 66% of all deaths in Germany in all age groups and both sexes, although the age-standardized death rates associated with these two CDs have been decreasing for more than ten years [7]. Another health challenge related to population aging is the fact that the number of people with multiple chronic conditions is increasing, leading to major changes in health care use and costs. In 2011, Lehnert and colleagues analyzed 35 studies investigating this relationship and demonstrated that the growing number of patients with multiple chronic conditions is associated with an increase in physician visits, hospitalizations, and the use of drugs [8]. 

Although Lehnert et al. underlined a number of problems related to health care utilization and costs in elderly people with multiple chronic conditions, their work only included two German studies performed in 1997 and 2000, and did not consider patients with only one CD. Therefore, the goal of our work was to evaluate the prevalence of defined CDs among older patients in German general practices (GPs) in 2013.

## Methods

### Database

The present study used data from the Disease Analyzer database (IMS HEALTH), which compiles drug prescriptions, diagnoses, as well as basic medical and demographic data obtained directly and in an anonymized format from computer systems used in the practices of general practitioners [9]. Diagnoses (ICD-10), prescriptions (Anatomical Therapeutic Chemical (ATC) Classification System), and the quality of reported data have been monitored by IMS based on a number of criteria (e.g., completeness of documentation, linkage between diagnoses and prescriptions). 

In Germany, the sampling methods used for the selection of physicians’ practices were appropriate for obtaining a representative database of primary care practices [9]. Prescription statistics for several drugs were very similar to data available from pharmaceutical prescription reports [9]. The age groups for given diagnoses in the Disease Analyzer also agreed well with those in corresponding disease registries [9]. 

### Study population

Patients older than 65 years who consulted a general practitioner between January and December 2014 were included. A total of 840,319 patients older treated by 1,243 general practitioners were available for analysis. 

Ten different CDs were considered based on primary care diagnosis (ICD-10 codes): hypertension (I10), lipid metabolism (E78), diabetes (E10-E14), coronary heart disease (I20-I25), cancer (C00-97), chronic obstructive pulmonary disease (J44), heart failure (I10), stroke (I63, I64, G45), osteoporosis (M80, M81), and chronic kidney disease (N18, N19). For these CDs, both new diagnoses and “status post” diagnoses were included.

### Statistical analysis

The present study only includes descriptive analyses. The number of patients older than 65 was calculated and the proportion (prevalence) of patients with defined CDs was estimated. The analyses were carried out using SAS version 9.3.

## Results

**Figure 1:**
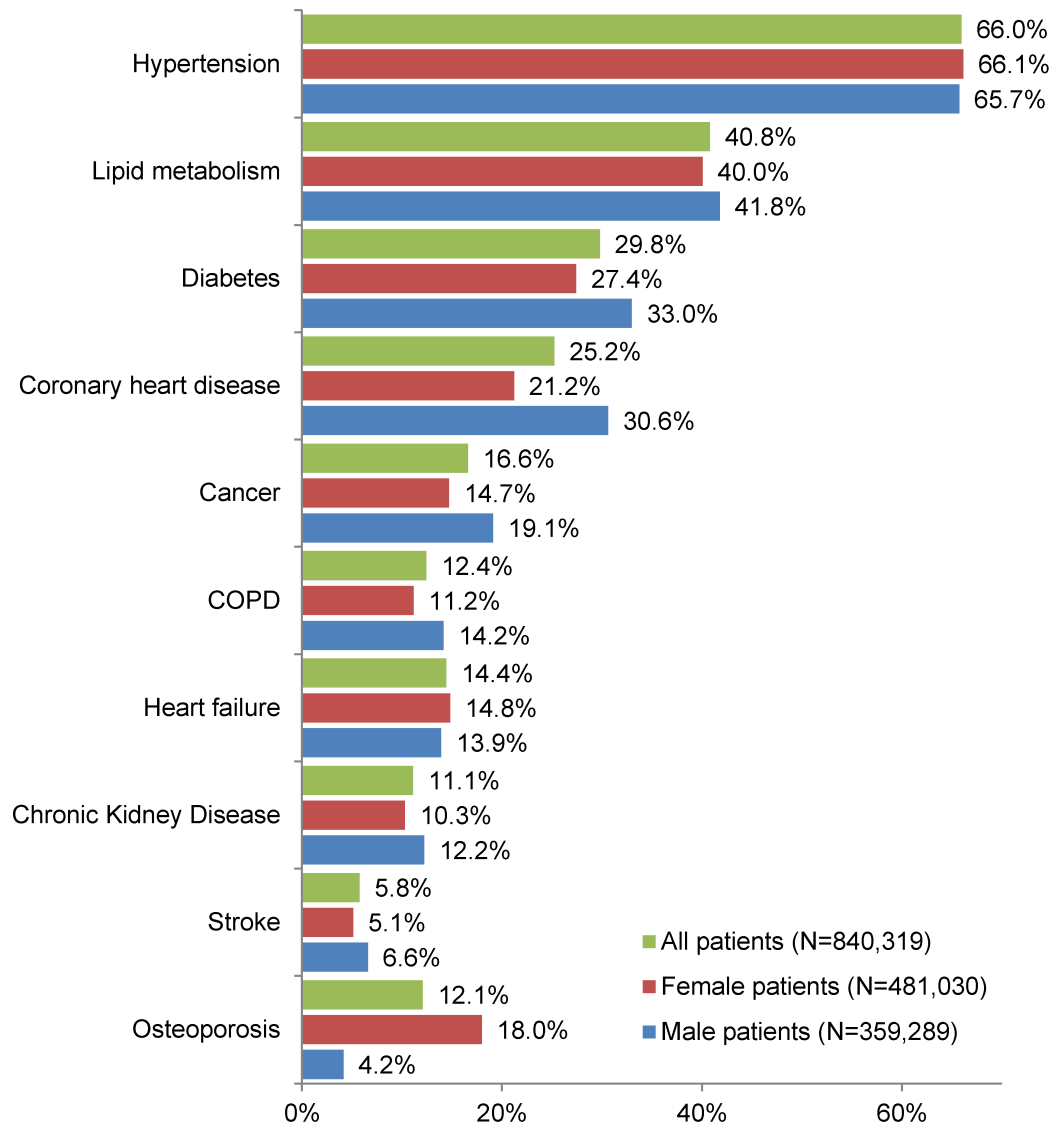
Prevalence of chronic diseases among patients over 65 years of age treated in German general practices

Figure 1 (Fig. 1) shows the prevalence of the ten selected CDs in men, women, and all patients over 65 years of age. The percentage of patients suffering from hypertension, lipid metabolism, diabetes, and coronary heart disease exceeds 20% in all three groups (men, women, and all patients). Hypertension was the most common CD, affecting 65.7% of men, 66.1% of women, and 66.0% of all patients. Stroke was the least common chronic condition, since it was diagnosed in only 6.6% of men, 5.1% of women, and 5.8% of all patients. 

Age and gender of older patients with chronic diseases are shown in Table 1 (Tab. 1).

**Figure 2:**
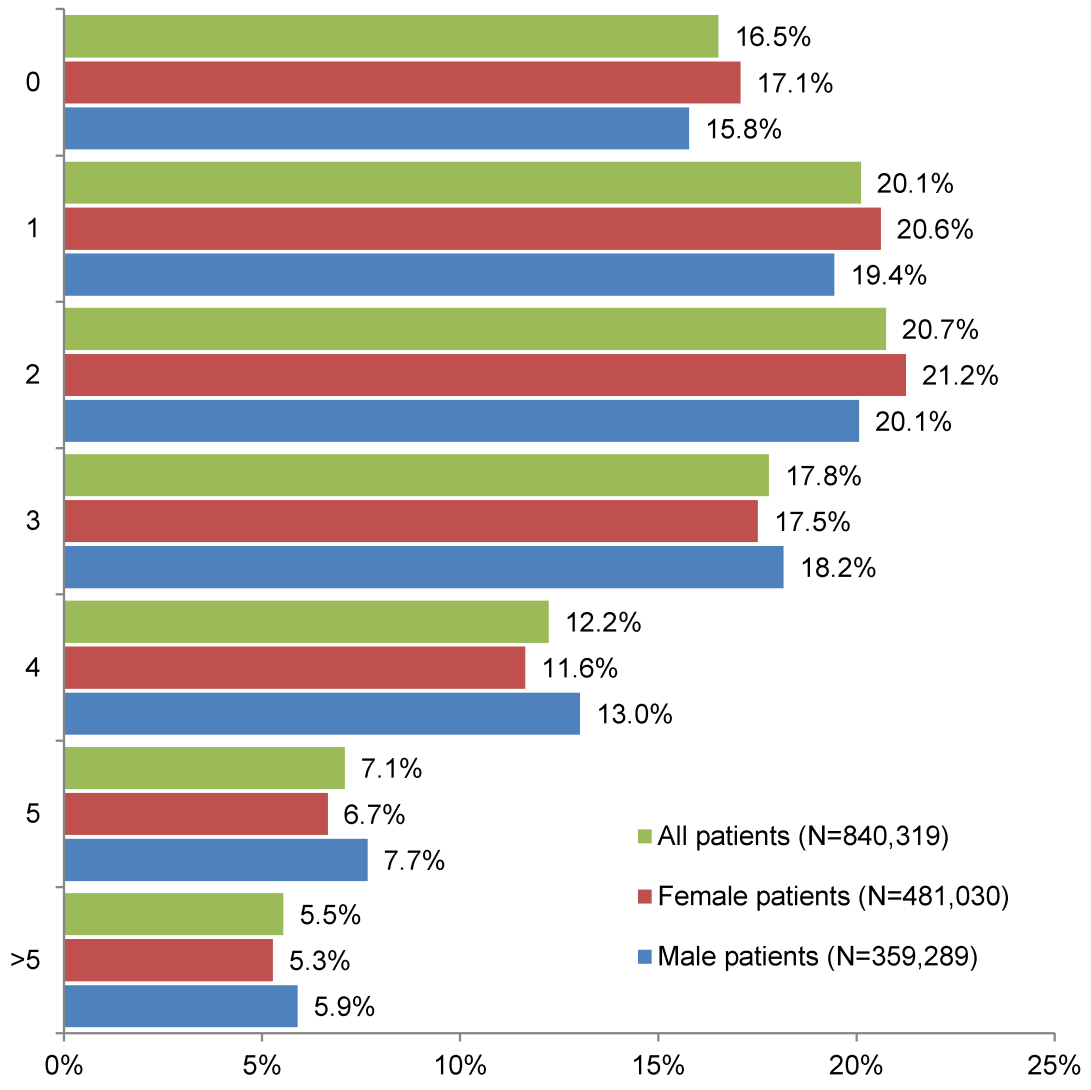
Number of different chronic diseases among patients over 65 years of age treated in German general practices

The number of different CDs among subjects over 65 years of age treated in German general practices is displayed in Figure 2 (Fig. 2). 15.8% of men, 17.1% of women, and 16.5% of all patients had no CD. By contrast, more than one out of two subjects had between one and three CDs (men: 57.7%, women: 59.3%, and all patients: 58.6%). Approximately 25% of subjects had four or more CDs (men: 26.6%, women: 23.6%, and all patients: 24.8%).

## Discussion

In this retrospective study, which included more than 800,000 German patients aged 65 years and over, we found that the prevalence of defined CDs was high. Hypertension and stroke were the most and the least frequent CDs, respectively. Interestingly, more than 50% of patients had between one and three CDs, and around 25% exhibited at least four CDs, underlying the prevalence of multiple chronic conditions in this population. 

Hypertension is known to be very common in the elderly. In 2003, Wolf-Maier et al. analyzed the prevalence of hypertension in Canada, the United States, and six European countries (Germany, Finland, Italy, England, Sweden, and Spain) [10]. Based on national surveys conducted in the 1990s, this work demonstrated that Germany had the highest prevalence of hypertension, ranging from 30%–40% in the age group 35–44 years to around 80% in the age group 65–74 years. The slightly lower rates found in our study may be explained by the fact that the prevalence in this study only include diagnoses of general practitioners what can lead to an underestimation of the prevalence of hypertension.

Lipid metabolism disorders were the second most common CD, with 41.8% of men and 40.0% of women affected by this disorder (40.8% of all patients). In Germany, the prevalence of dyslipidemia was studied in 2008 by Steinhagen-Thiessen and colleagues [11]. In fact, these authors found that the proportion of patients in primary care practices with lipid metabolism disorders increased up until the age of 70 (87.7% of men and 92.7% of women in the age group 61–70 years), although this proportion decreased in older patients (61.1% of men and 86.1% of women in the age group 91–100 years). Even though this late decrease was not discussed by Steinhagen-Thiessen et al. [11], one can easily imagine that it is related to the low number of patients included in the age group 91–100 years, which consisted of just 18 men and 36 women compared to 2,718 men and 3,397 women in the age group 61–70 years. Furthermore, it is also important to bear in mind that the prevalence of dyslipidemia was found to be higher in this work than in our study or in others performed in different areas of the world [12], [13], [14]. This slight discrepancy may be explained by the fact that the methods chosen by the authors and the countries of interest were not identical. 

The prevalence of diabetes, coronary heart disease, cancer, chronic obstructive pulmonary disease, heart failure, chronic kidney disease, stroke, and osteoporosis ranged from 4.2% (osteoporosis) to 33.0% (diabetes). These results are in line with the literature. In 2003, Jain and Paranjape found that among 585 elderly people, around 30% suffered from type 2 diabetes [15], which corroborated previous works [16]. In the case of coronary heart disease, the percentage of patients was slightly lower (30.6% in men and 21.2% in women). As a matter of fact, similar data were found in a national survey conducted between 2009 and 2012 (men: 32.2%, women: 18.8% in the >80 years group) [17]. By contrast, we found a higher prevalence of cancer than a previous study performed in 2003 in the United Kingdom [18]. Nonetheless, it is important to remember that the studies of Forman et al. used methods different to ours and were conducted in different countries. Finally, the prevalence of chronic obstructive pulmonary disease, heart failure, chronic kidney disease, stroke, and osteoporosis was approximately less than 15% among the elderly in both men and women, confirming previous findings [17], [19], [20], [21], [22]. 

Furthermore, one important result of our study is that around 25% of patients had more than 4 CDs. The presence of multiple chronic conditions results in serious health consequences, since persons with several CDs are more limited than persons with only one chronic condition [23]. Furthermore, it has been suggested that there are some disease-disease interactions, which may increase the adverse effects of CDs on patient disability. Another major consequence of multiple chronic conditions is that patients use different health services and are at a higher risk of suboptimal quality care [23]. Indeed, it has been found in Medicare that the number of different physicians seen per year and per patient ranges from 4 when there is only one CD to 14 when there are more than 4 CDs [23]. Finally, people with several chronic diseases are also an economic challenge, as the cost related to their treatment and their management is particularly high [24]. As a matter of fact, per patient Medicare expenditures increased with the number of chronic conditions from $211 among subjects without a chronic condition to $13,973 among subjects with at least 4 CDs [24]. These findings have been corroborated in the recent review of Lehnert et al., summarizing 35 different studies [8].

This study was subject to several limitations. The assessment of diagnoses relied solely on ICD codes by primary care physicians. It should be mentioned, that GPs most likely did not use universal criteria for defining hypertension, dyslipidemia, or other chronic diseases.

Data on socioeconomic status and lifestyle-related risk factors were also unavailable. Only ten diagnoses were analyzed since other, different, diagnoses were not included. Moreover, the number of different CDs per patient is based on these ten CDs only. 

However, the strength of the study is the large nationwide database used and the unbiased assessment of diagnoses.

To summarize briefly, our study showed that the prevalence of CDs is high in the German elderly population. Hypertension was the most frequent chronic condition and around 25% of patients exhibited at least four CDs. 

## Data

Data for this article are available from the Dryad Repository: http://dx.doi.org/10.5061/dryad.qh0h1 [25].

## Notes

### Competing interests

Karel Kostev is an employee of IMS Health. IMS Health (http://www.imshealth.de/sites/en/about-us/our-company) is a commercial research institute providing information, services and technology for the healthcare industry. Louis Jacob and Jessica Breuer declare that they have no competing interests.

## Figures and Tables

**Table 1:**
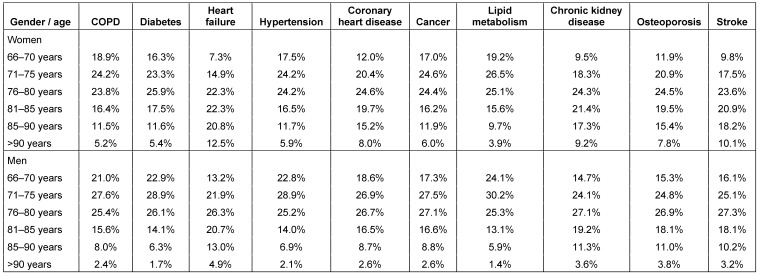
Age and gender of older patients with chronic diseases in German general practices
